# *“It is good to take her early to the doctor” –* mothers’ understanding of childhood pneumonia symptoms and health care seeking in Kilimanjaro region, Tanzania

**DOI:** 10.1186/s12914-017-0135-1

**Published:** 2017-09-22

**Authors:** Florida Muro, Judith Meta, Jenny Renju, Adiel Mushi, Hilda Mbakilwa, Raimos Olomi, Hugh Reyburn, Helena Hildenwall

**Affiliations:** 1 Kilimanjaro Christian Medical University College, P.O.Box 2240, Moshi, Tanzania; 2 Kilimanjaro Christian Medical Centre, P.O.Box 3010, Moshi, Tanzania; 3 Joint Malaria Programme – Kilimanjaro Christian Medical Centre, P.O.Box 2228, Moshi, Tanzania; 4 London School of Hygiene and Tropical Medicine (LSHTM), Keppel St, London, WICE7HT UK; 5 The National Institute for Medical Research (NIMR), 3 Barack Obama Drive, P. O. Box 9653, 11101 Dar es Salaam, Tanzania; 6 Department of Public Health Sciences, Global Health – Health System and Policy Research Group, Karolinska Institutet, SE-171 77 Stockholm, Sweden

**Keywords:** Acute respiratory illness (ARI), Care-seeking, Children, Fever, Illness concepts, Pneumonia

## Abstract

**Background:**

Pneumonia is among the leading causes of avoidable deaths for young children globally. The main burden of mortality falls on children from poor and rural families who are less likely to obtain the treatment they need, highlighting inequities in access to effective care and treatment. Caretakers’ illness perceptions and care-seeking practices are of major importance for children with pneumonia to receive adequate care. This study qualitatively explores the caretaker concepts of childhood pneumonia in relation to treatment seeking behaviour and health worker management in Moshi urban district, Tanzania.

**Methods:**

In May - July 2013 data was gathered through different qualitative data collection techniques including five focus group discussions (FGDs) with mothers of children under-five years of age. The FGDs involved free listing of pneumonia symptoms and video presentations of children with respiratory symptoms done, these were triangulated with ten case narratives with mothers of children admitted with pneumonia and eleven in-depth interviews with hospital health workers. Transcripts were coded and analysed using qualitative content analysis.

**Results:**

Mothers demonstrated good awareness of common childhood illnesses including pneumonia, which was often associated with symptoms such as cough, flu, chest tightness, fever, and difficulty in breathing. Mothers had mixed views on causative factors and treatments options but generally preferred modern medicine for persisting and severe symptoms. However, all respondent reported access to health facilities as a barrier to care, associated with transport, personal safety and economic constraints.

**Conclusion:**

Local illness concepts and traditional treatment options did not constitute barriers to care for pneumonia symptoms. Poor access to health facilities was the main barrier. Decentralisation of care through community health workers may improve access to care but needs to be combined with strengthened referral systems and accessible hospital care for those in need.

## Background

Pneumonia is the biggest single cause of death among children under the age of five years outside the neonatal period, accounting for an estimated 15% of all deaths in this age group worldwide [1]. These deaths present as one of the most serious global public health challenges faced by the international community, nevertheless, pneumonia deaths can largely be averted if appropriate antibiotics and supportive treatment are given early. Timely caregivers recognition of pneumonia symptoms, appropriate care-seeking and correct management by a health care provider remains the crucial step to reduce mortality [2].

A recent UNICEF report states that 55% of caregivers sought appropriate care for children with suspected pneumonia although only 39% of children received antibiotics [3]. According to a recent Tanzania Demographic Health Survey data, 4 % of the under-five children were reported to have respiratory symptoms two weeks prior to the survey of which 55% sought care at a health facility or provider. [4] While this may highlight a need for improved quality of facility care it also implies that many children with potential pneumonia remain untreated as a result of failure in seeking and accessing care [5, 6]. In Tanzania, children from the poorest families are often the most susceptible to childhood illnesses including pneumonia in part because access to correct care in facilities is inadequate [7]. The Integrated Community Case Management (iCCM) strategy (advocated by the WHO/UNICEF) is purported to promote equity, local care-seeking and contributes to the reduction in child mortality by improving access and treatment availability at the community level [8, 9]. Earlier studies have demonstrated a role of local illness concepts coupled with limited community awareness of pneumonia symptoms as potential barriers to timely healthcare seeking along with gender, financial, and logistical barriers [10].

In recent years’, global health initiatives have focused on promoting increased access to care and treatment in the communities. Still, an unacceptably high mortality persists with many children remaining undiagnosed and/or untreated. The aim of this study was to explore the local perceptions of pneumonia symptoms and the related care-seeking actions to identify potential barriers for adequate pneumonia management.

## Methods

### Study area and population

The study was conducted in Moshi municipality (population is approximately 184,292), in Kilimanjaro region in northern Tanzania (2012 census) [11]. The predominant ethnic groups are Chagga and Pare. Key economic activity in the region is farming, trading of primary produce, small retail outlets and tourism. Ninety percent of the population is living within five kilometres from a network of health facilities including Kilimanjaro Christian Medical Centre which serves as the zonal referral hospital, Mawenzi regional hospital and St. Joseph’s designated municipal council hospital as well as eight health centres and 47 dispensaries [12].

Since 2002 Tanzania initiated a “no fee health service policy” for children under five year of age and antenatal care in order to promote equitable health care [13]. According to the Tanzanian Demographic Health Survey of 2015/16, the under-five mortality rate is 67 deaths per 1000 live births [4].

### Data collection

Different methods were used to collect qualitative data from May to July 2013. Focus group discussions (FGDs) with mothers of children under-five years of age were triangulated with four data collection techniques adapted from the Focused Ethnographic Study (FES) protocol, as recommended by the WHO [14]. The FES techniques included: 1) free listing to elicit specific terms of pneumonia and associated symptoms by guardians; 2) presentation of a video showing children with respiratory symptoms to understand mother’s perception of these symptoms; 3) narratives of recent pneumonia episodes from mothers with children under-five years; and 4) in-depth interviews with health care providers about their perceptions of mothers’ management of acute respiratory infection (ARI).

Data was collected by the first and second author (FM and JM) who are both fluent in Chagga and familiar with the culture of the studied community: FM is a medical doctor and public health researcher while JM is a social scientist. One author was moderating while the other one took notes interchangeably.

Participants included purposively selected health care workers (HCWs) and mothers of children under-five years. Commonly mothers are the main caretaker of young children and therefore deemed as the most important informant regarding health problems in young children [10]. Additionally, only mothers were included to ensure a homogenous group in terms of the relationship to the child.

#### Focus group discussions

Five FGDs were held with a total of 43 mothers over five weeks’ time. The mothers were divided into different groups depending on their age (2 groups with mothers 16-25 years old and 3 groups with mothers 26-40 years old) to encourage comfortable discussion as women with similar characteristics or experiences may share views with each other more openly. Each FGD included eight to ten mothers’ and lasted between 90 and 120 min. Eight out of thirty four villages in Moshi Municipality were randomly selected using a lottery method. With the support of the village chairman, all the households with children under-five years were listed, and the list included their mothers’ age to enable purposeful selection of mothers according to their age (young and older separately). Twelve mothers were selected from each village for invitation to participate in the FGDs. The FGD guide focused on exploring mother’s understanding and perceptions of common childhood illness, the cause of illness and care-seeking preferences.

Each FGD included a video presentation at the end showing children with different respiratory symptoms of pneumonia to determine how mothers perceived these symptoms (Table 1).

**Table 1:** Presenting symptoms for the three video scenarios showing different levels of pneumonia severity as per WHO definitions

Video Scenario	Child Symptoms
1	Five month old child with chest in-drawing and fast breathing
2	Three month old child who was snoring and coughing
3	One year old child with chest indrawing, wheezing, groaning and fast breathing

#### Case narratives

Ten case narratives were conducted with mothers of children under-five years of age that had recently been admitted and treated for pneumonia at St Joseph hospital. Mothers were consecutively recruited upon discharge at St. Joseph’s hospital after being given study information and written consent. Those who consented were interviewed in their home within two weeks from discharge and the interview took 45-60 min.

The narratives sought to understand how mothers recognized and responded to their child’s illness episode including action taken at home, treatment seeking options and barriers faced.

#### In-depth interviews

Over four weeks, eleven In-Depth Interviews (IDIs) were held with HCWs at St Joseph’s hospital including one medical doctor, assistant medical officers (3), clinical officers (2) and nurses (2) and medical aids (3). IDIs were directed by topic guides and conducted to obtain their views regarding community perceptions and subsequent practice in regard to paediatric pneumonia symptoms in children under-five years of age. HCWs were purposefully selected paediatric out-patient department and/or ward and were included if available during the study period and willing to provide a written informed consent to participate.

### Data management and analysis

FGDs and individual interviews were recorded using an MP3 device and the audio files were transferred to a project server after completion of interviews. The audio files were transcribed into Swahili which were then translated into English and checked for accuracy through back-translation. FM compiled all the data including the field notes and developed a list of codes that were discussed with co-authors (JR and HH). Any illness terminology remained in the final transcript in either Swahili or the local dialect Chagga.

Manual analysis was conducted using latent content analysis [15]. Three authors (FM, JR and JM) coded matching data into different sub themes to allow coherent presentation of the captured issues. Coding started by identifying relevant information’s from all transcripts, followed by coding such information into matching categories and semi-categories which were compared and contrasted, then collapsed into major themes and sub themes.

## Results

### Background characteristics of the study participants

The mean age of mothers was 28.7 (SD ± 6.6) years and most (42/53) were aged between 20 and 35 years. The majority of included mothers (39/53) had completed primary education and 14/53 had attended secondary school or above. Most participants were married (44/53) and engaged in small-scale business (35/53), farming (8/53) or were house-wives (10/53). The majority of mothers (50/53) had only one child less than five years of age.

Of the interviewed health workers, 5/11 had been trained in IMCI and the majority (8/11) had practiced for more than three years.

### Local illness terminologies

Mothers listed several common childhood illnesses during the free listing sessions and in FGDs. These included literate translations of pneumonia, malaria, diarrhea and urinary tract infections. Most mothers mentioned cough (*kikohozi*), rib (chest) tightness (*mbavu kubana*), difficulty in breathing (*kuhema/kupumua kwa shida*) and wheezing/snoring (*sauti ya mkwaruzo/kukoroma*) as commonly observed symptoms of pneumonia.

“*The first symptom (of pneumonia) is that the child will cough a lot but sometimes there is no coughing but you will see how she is short of breath and you will know clearly that the chest is tight.”* (FGD, young mother).

Fast breathing (*kuhema haraka haraka*) and chest in-drawing (*mbavu kuvuta*) were rarely mentioned symptoms until after further probing. Other signs and symptoms associated with pneumonia were fever (*joto/kuchemka/wa moto*), flu/running nose (*mafua*) diarrhea (*kuharisha*), and vomiting (*kutapika*).

They term chest tightness (*mbavu kubana*) was noted as a symptom for both pneumonia (*kifua cha kubana na mbavu*) and part of asthma (*pumu*).

*“... most women understand lemonia (pneumonia) as mbavu kubana (chest tightness), until unable to breath then realises it is lemonia; Another one who does not understand at all says its pumu (asthma).”* (Female HCW).

Other words used for respiratory symptoms were *kifua*, which literally translates as chest and was used for both mild and sometimes severe chest illness interchangeably. Mothers named a non-severe cough as *kifua* while a cough with another sign such as chest tightness or in-drawing (more severe) was now regarded as *kifua cha pneumonia*.

*“Unlike normal kifua (chest) which implies that you just cough; ‘kifua cha pneumonia’ tightens the ribs (kinabana mbavu).” (FGD, older mothers).*

*Kirumu* emerged as a term involving a severe chest problem with difficulty breathing in children. It was said by a few mothers that *kirumu* could present with cough, sometimes also a congested chest, difficulty in breathing and noisy breathing (*kru kru* sound). Some mothers in the case narratives and FGD video sessions associated *kirumu* to a chronic or recurrent chest illness.

*“Kirumu - they say it’s when a child’s chest tightens then you take him/her to hospital; the first time s/he is given medicine and recovers, the second time s/he snores a lot at night when s/he sleeps and an area in her/his stomach here [sign] if s/he breaths it enters in.” (FGD older mothers).*

*“…. but when the fever goes higher, the chest starts tightening, as it tightens the chest snores as the child snores as if kohozi (sputum) wants to get out but not, therefore you are told this is kirumu.” (Case narrative).*

Mothers raised mixed opinions when discussing the video that depicted a child with chest indrawing, wheezing and fast breathing (Table 1). Some argued that it was severe illness requiring immediate hospital care, a few identified it as *kirumu,* and therefore not requiring hospitalization. Its recurrent nature led some mothers to believe that hospital care was not effective. Some went further to state that local treatment by a particular elderly woman was required to cure the child.

*Kilimi* (the uvula) was another term used by mothers to describe an illness that could cause cough and was also perceived to be a potential cause of pneumonia symptoms in children.

*“*W*hen kilimi (the uvula) has grown big and it is swollen there is discomfort when a child tries to swallow something, so s/he coughs.” (FGD, young mothers).*

### Perceived cause and severity

Pneumonia symptoms were reported by mothers to be caused by a variety of factors including exposure to cold (weather, meals and drinks), germs like bacteria, overheating, smoke and swelling of the uvula (*kilimi)*.

*If a child has nimonia (pneumonia), it can be germs causing it…. I think bacteria, they tell us in clinic (FGD, young mothers).*

*There’s a certain nurse, she is a neighbour.... “when I told her my child’s chest (problem) like this and this, she said he must have an infection.” (Case narrative).*

*“Smoke from burning charcoal and fathers or mothers who are cigarette smokers can also be the cause especially if they do so in the houses or close to children.” (FGD, older mothers).*

Unlike pneumonia which mothers attributed to cold exposure, *kirumu* was said to sometimes be a chronic condition caused by swallowing of amniotic fluid during birth.

“… *So they are two different things, kirumu is when a child swallows dirty water during birth while nimonia is a chest [infection] that the child gets after exposure to cold if you have not covered her with a sweater during a cold period.” (FGD, older mother group).*

Others believed respiratory symptoms were caused by eating or drinking cold things when pregnant while others attributed it to environmental or seasonal changes such as during maize flowering, sighting of new moon or a change in weather.

### Household prevention and treatment practices

Several remedies were mentioned for managing respiratory symptoms through first aid. Remedies like honey, lemon, eggs, milk, garlic, ginger and bicarbonate soda were used to treat cough and flu, fever or chest congestion.

“*With my knowledge, when the chest problem starts I look for a raw egg, break it and give it to the child, it helps.*” *(FGD, older mothers).*

Apart from home remedies, it appeared that mothers tried home treatment with pharmaceutical products based on previous prescriptions from health facilities. Mothers expressed awareness with administering cough syrups and antipyretics. They would take the child to the hospital if the conditions persisted or symptoms were perceived to be severe.

*“*S*ometimes if you see such a condition [cough] you give her sodium bicarbonate and lemon and when morning comes if you remember what medicine she takes you go to the pharmacy to buy it and give it to her. If you see that it is not helping then you have to go to hospital and arrange for chest examination so as to know what medicine you will use.” (FGD, older mothers).*

This practice of using biomedical drugs at home was described by HCWs and also supported by case narrative whereby mothers described how they would administer a few doses of antipyretics bought during the current illness or left over from previous illness episodes. If the fever persisted or they noticed additional symptoms such as shivering and an inability to breastfeed or play they consulted a health care provider.

*“Home treatment is common. You find that you have received a child, the mother tells you s/he has already taken such and such drug(s), I bought them at pharmacy after seeing that a child has this problem.”. (IDI, male HCW).*

### Care seeking and decision-making

Several mothers reported to make decision on the choice of treatment based on the perceived severity of pneumonia symptoms. In that regard, the majority of respondents reported personal and broader community experiences of seeking care from medical practitioners for children with perceived severe, danger or persistent respiratory symptoms.

*“It is good to take her early to the doctor so you know how her health condition progresses. Not just to give her (home treatment) and continue keeping her at home, you might endanger the health condition of the child.” (FGD, young mothers*).

Danger signs or symptoms such as inability to breastfeed or weakness (*legea*) and fever not responding to first aid drove mothers to seek outside care.

*“I knew my child has lemonia (pneumonia), he was weak (legea), unable to breastfeed, breathing with difficulty and unusual breathing (eh, eh); lemonia has to be taken to specialists who are at hospitals.” (Case narrative).*

The majority of mothers mentioned that decisions to seek care were taken by themselves. However, they reported to also seek advice or help from the father, neighbour or pharmacist before rushing to hospital, and this was well supported by HCWs.

*“…most of the time I think first, I go to a nurse in the neighbourhood to seek advice. If she has some medication she gives me. When the condition doesn’t improve, I go to hospital.” (FGD, young mothers).*

*“Many times, until coming to hospital you find that the mother alone took the initiative, you often find the mother coming alone, she explains her problem and if you look at how the child is, you just help.”* (IDI, male HCW).

Mothers in case narratives reported that their children had been very sick but only few were aware that it was severe pneumonia unless told by others like family members, neighbours or health workers.

*“Even when I took him to hospital everyone kept saying that my child isn’t breathing well, even in the streets. He breathes [shows sign], it’s like when he wants to breathe his chest sinks down again.” (Case narrative).*

Whilst some mothers reported to consult an elderly woman (bibi) providing traditional care, traditional healers were overall not a common source of care. Mothers reported that traditional healers were used in the past and nowadays they have shifted to modern care. HCWs confirmed this by suggesting that only few mothers, if any at all, seek traditional treatment.

*“*A*ah in my opinion it’s only a few, very few (mothers) who goes to get those medicines (traditional) but most of them in a large proportion they come to seek care at hospital.”* (IDI, male HCW).

Time of illness occurrence emerged as a potential barrier to immediate care seeking from a health facility. Often mothers in FGDs and those with a recent experience shared their concern particularly of security and transport difficulties if illness symptoms started at night times.

*“You know, when I just see body temperature rising (moto) I give him [panadol] so it stabilizes him, so it reaches time to take him to hospital, as here you cannot pass alone to go to dispensary.” (Case narrative).*

Poverty was another factor reported to inhibit timely care seeking. For example, cost of treatment determined whether care should be started at home or sought outside the home.

*“We would generally try something at home first before [going to the] hospital, especially if there is no money available.” (FGD, young mother).*

*“Often, like now the drugs are not free as the government has promised, most of times they are sold. It’s not a secret but these other costs like a bed, may-be a drip, those you will not be charged for, but drugs you must pay straight.”* (IDI, male HCW).

## Discussion

While global child mortality has declined during the past years the numbers of avoidable deaths among young children in resource poor settings is still unacceptably high and children continue to die from treatable causes [16]. Local illness concepts and the use of traditional care have been reported as a potential barrier for adequate care for children with infectious diseases [17–20]. However, in this study we report on a community understanding of the medical concept of pneumonia, maternal awareness of pneumonia severity symptoms and an overall preference for rapid care at health facilities for a child with suspected pneumonia. The main barriers for prompt and adequate pneumonia care were related to access issues involving treatment associated costs, transport to health facilities and fear for their personal safety when seeking care at night.

In line with a similar study from low-income settings, most mothers referred to symptoms of conditions rather than the actual disease [20]. Fast breathing and chest in-drawing were rarely mentioned as commonly recognized symptoms compared to cough or difficulty in breathing and fever. However, when presented with children with these symptoms in video presentations, all mothers agreed on the need to seek immediate facility care for the child. While chest in-drawing is many times the primary manifestations of difficulty breathing it is possible that both chest in-drawings and fast breathing are embedded in the overriding concept of difficulty in breathing [20, 21].

Mothers displayed a good awareness of pneumonia and were even aware of some potential risk factors for respiratory problems such as aspiration of dirty amniotic fluid during delivery and exposure to smoke [22]. This is contradicting previous studies from Tanzania and elsewhere that have pointed at a tendency of caretakers to believe all fevers are due to malaria [20, 23]and disparities between medical diagnoses in hospital and perceived causes of admissions [24]. The respondents in our study were relatively well educated (73.8% attained primary level education while 26.2% had secondary level education or above), previous studies have shown that understanding health and ill health can improve with higher levels of education [25–27]. Further, there has been an increased use of rapid diagnostic tests for malaria in our study area. Improved access to and accuracy of diagnosis could have positively affected the caretakers’ understanding of fever, thereby reducing the tendency to label all fever as “malaria”. Mothers in our study even mentioned bacteria or viruses as causes of pneumonia. This is also in contrast to other studies where a limited community understanding of pneumonia aetiology has been shown [28–31]. An awareness of causes and risk factors may contribute to improved child health through the application of preventive and control measures. However, antibiotics were rarely reported to be used at home. Mothers were aware of antibiotics but expressed limited experience of handling these drugs at home as they were perceived to be meant for hospital care. While home treatment with antibiotics has been reported from a variety of studies [21, 32–36], a restricted use of antibiotic is important to limit the progress of antibiotic resistance.

Traditional care was generally reported to belong to the “old days”. The concept of *kirumu* emerged as the only condition that for some respondents could pose a risk for treatment seeking delays. The described presenting symptoms for *kirumu* resemble the symptoms present in severe pneumonia or asthma/bronchiolitis and were reported to be a recurrent or chronic condition for which biomedical treatment was not always considered preferable except when the child’s condition becomes serious. This finding however contradicts a study of illness concept were the perceived treatment needs change from biomedical to traditional as a child’s symptoms move from mild to severe and concurrently are perceived to be more “suspicious” [37].

Also in contrast to previous studies mothers reported very limited use of traditional healers and health workers confirmed this [17, 23]. Most women reported to rely on biomedical medicine or seeking care at health facilities as traditional medicine was perceived to belong to the “old days” [38]. However, as with other settings, there was reportedly a frequent use of middle layers between home and hospital and these may delay appropriate care at facility as a result of unsuccessful attempts to treat with home remedies [21, 39].

It is worth emphasizing that the included women were relatively well educated which could explain their good understanding of illness. They also live in an area with an acceptable access to health care providers within 5 km from their homes. Mothers furthermore proved independent from husbands and relatives in making decisions regarding the care-seeking for their child. Similar findings were noted in Ghana and Guinea whereby caregivers were reported to have the power to seek any form of care and act quickly [40, 41]. Conversely other studies reported that mothers had limited or no power to make decisions freely for their child’s care outside the home regardless of their financial status in the household. In these settings, the mothers were reported to still require permission from their husbands or head of household and sometimes their mother-in-law’s [21, 42–44].

Evidence of an increased acceptance of a mother’s autonomy in decision making concerning their child’s health and the subsequent health seeking behaviour could be indicative of the increased empowerment of women and perhaps increased gender equality. This may have a huge impact for improved child health [40, 45].

Despite these apparently favourable circumstances, a number of issues still contributed to potential delays to adequate care seeking, the main constraint relating to financial, independence, personal safety and access to transport to reach the health facilities. Notwithstanding the policy of free care for young children in Tanzania, mothers in this study cited costs associated with visiting a health facility including transport and purchasing of medicines as barriers to prompt care seeking. In similar studies, such factors have been shown to influence caretakers to seek alternative care such as traditional treatment [46, 47]. Our results suggest that improved access to care should be made a priority to reduce avoidable child deaths in the study area. Integrated Community Case Management (iCCM) programs have the ability to improve access and have been shown to reduce the burden of pneumonia [8, 48]. Still, a certain proportion of children will continue to develop severe disease and require hospital care while access also needs to be improved through strengthening of referral systems and provision of emergency care.

### Limitation

We triangulated data from FGDs, IDIs and case narratives, used different informants (mothers and health care workers) as a way to confirm consistency and potential disagreement between views of respondents. The use of video presentation of sick children revealed mothers’ perceptions and preferred actions on respiratory symptoms. However, since no observations were performed, the methods used reveal people stated perceptions and actions rather than their actual action. The generalizability of findings is limited since illness concepts are context specific.

## Conclusion

Our findings demonstrate a good community understanding of childhood pneumonia symptoms with a strong preference for modern medicine and facility care. However, access to appropriate care was limited due to cost of transport and care along with transport difficulties and especially so for care seeking attempts during evenings and nights. These community and formal health service delivery barriers need to be addressed through improved referral systems along with efforts to providing healthcare services such as integrated community case management programs nearer to the communities.

## Abbreviations

ARI: Acute Respiratory Illness
FES: Focused Ethnographic Survey
FGD: Focus Group Discussion
iCCM: Integrated Community Case Management
IDI: In-Depth Interview
UNICEF: The United Nations Children’s Fund
WHO: World Health Organization
